# Identification of diagnostic molecules and potential traditional Chinese medicine components for Alzheimer’s disease by single cell RNA sequencing combined with a systematic framework for network pharmacology

**DOI:** 10.3389/fmed.2023.1335512

**Published:** 2024-01-05

**Authors:** Tao Wang, Xinlei Zhang, Wenxin Liu, Fangli Ning, Xingling Hu, Lei Qin, Mengmeng Cui, Jinyue Yang, Shi Lv, Qian Wang

**Affiliations:** ^1^Neck-Shoulder and Lumbocrural Pain Hospital of Shandong First Medical University, Shandong First Medical University, Jinan, China; ^2^Department of Rehabilitation, The Second Affiliated Hospital of Shandong First Medical University, Taian, China; ^3^Department of Neurology, The Second Affiliated Hospital of Shandong First Medical University, Taian, China; ^4^Department of Central Laboratory, The Affiliated Taian City Central Hospital of Qingdao University, Taian, China

**Keywords:** Mendelian randomization, network pharmacology, single-cell transcriptome sequencing, traditional Chinese medicine, Alzheimer’s disease

## Abstract

**Background:**

Single-cell RNA sequencing (scRNA-Seq) provides new perspectives and ideas to investigate the interactions between different cell types and organisms. By integrating scRNA-seq with new computational frameworks or specific technologies, better Alzheimer’s disease (AD) treatments may be developed.

**Methods:**

The single-cell sequencing dataset GSE158234 was obtained from the GEO database. Preprocessing, quality control, dimensionality-reducing clustering, and annotation to identify cell types were performed on it. RNA-seq profiling dataset GSE238013 was used to determine the components of specific cell subpopulations in diverse samples. A set of genes included in the OMIM, Genecards, CTD, and DisGeNET databases were selected as highly plausible AD-related genes. Then, ROC curves were created to predict the diagnostic value using the significantly expressed genes in the KO group as hub genes. The genes mentioned above were mapped to the Coremine Medical database to forecast prospective therapeutic Chinese medicines, and a “Chinese medicine-ingredient-target” network was constructed to screen for potential therapeutic targets. The last step was to undertake Mendelian randomization research to determine the causal link between the critical gene IL1B and AD in the genome-wide association study.

**Results:**

Using the scRNA-seq dataset, five unique cell clusters were discovered. These clusters were further subdivided into four distinct cell types using marker genes. The KO group showed a more substantial differential subgroup of macrophages than the WT group. By using the available datasets and PPI network analysis, 54 common genes were discovered. Four clusters were identified using the MCODE approach, and correlation analysis showed that seven genes in those four clusters had a significantly negative correlation with macrophages. Six genes in four sets had a significantly positive correlation. Five genes had different levels of expression in the WT and KO groups. The String database was used to identify the regulatory relationships between the four genes (IL10, CX3CR1, IL1B, and IL6) that were finally selected as AD hub genes. Screening identified potential traditional Chinese medicine to intervene in the transformation process of AD, including Radix Salviae, ginseng, Ganoderma, licorice, Coptidis Rhizoma, and Scutellariae Radix, in addition to promising therapeutic targets, such as PTGS1, PTGS2, and RXRA. Finally, it was shown that IL1B directly correlated with immune cell infiltration in AD. In inverse variance weighting, we found that IL1B was associated with a higher risk of AD, with an OR of 1.003 (95% CI = 1.001–1.006, *p* = 0.038).

**Conclusion:**

Our research combined network pharmacology and the scRNA-seq computational framework to uncover pertinent hub genes and prospective traditional Chinese medicine potential therapeutic targets for AD. These discoveries may aid in understanding the molecular processes behind AD genes and the development of novel medications to treat the condition.

## Introduction

The world’s population structure is changing due to increased life expectancy. Even though lifespans are increasing, not everyone is in excellent health. The current population has around 50 million dementia sufferers, the bulk of whom are elderly. By 2040–2050, 100–130 million people are projected to have dementia, with Alzheimer’s disease (AD) accounting for over 70% of these cases (1, 2). AD is a neurological illness that progressively worsens and impairs memory. Despite much scientific and clinical study, the prevention and treatment of AD have not advanced much (3). If effective treatments are not developed, AD might become a global Epidemic.

Typical tissue pathology characteristics of AD are abnormal primary fiber sedimentation of starch-like protein β (Aβ) and neurogenic fiber bonding of tau protein (4). In recent years, it has been shown that neuroinflammation and tissue-resident immune cells are critical participants in the pathogenesis of AD (5, 6). Brain-resident microglia are an essential component of the localized immune system in the central nervous system (CNS) and are closely related to the genetics and neuropathology of AD (7). Microglia, the brain’s primary immune cells, perform essential macrophage functions, including phagocytosis of protein aggregates and dead cell debris, cytokine/chemokine signaling, immunological response, and surveillance. Microglia also carry out crucial neuroprotective parts, including trophic support for neurons, oligodendrocyte differentiation stimulation, and pruning and plasticity management of synaptic connections (8). With the recent identification of several AD risk locations near the immune gene, combined with the development of polyvalent stem cell-derived microglial cells and embedded mouse models, it is increasingly possible to study the mechanisms for the impact of human microglia cells on AD risk. A study suggests that cell therapy with stem cells may be therapeutically effective in preventing the pathogenesis of AD. Although many strategies have focused on using stem cells to regenerate damaged neurons, new studies have demonstrated stem cells’ immunomodulatory function, which regulates microglia’s activity state and mediates neuroinflammation. Thus, understanding the molecular mechanisms involved in brain homeostasis through the protective characteristics of mesenchymal stem cells (MSCs) could lead to remedial treatments for AD (9). Moreover, human-induced pluripotent stem cell-derived microglia are strongly correlated with the inheritance of Alzheimer’s disease and significant neuropsychiatric disorders (10). These studies have important implications for the discovery of AD mechanisms. Variants in the triggering receptor expressed on myeloid cells 2 (TREM2) had the highest effect on disease risk among these microglia-specific AD risk loci; the elevated risk is comparable to that of an APOE ε4 allele (11). Since microglia are the primary source of TREM2 expression in the central nervous system, this raises significant concerns regarding TREM2’s function in promoting microglia-specific processes linked to neurodegenerative disorders. TREM2, a member of the immunoglobulin superfamily, mediates responses to phospholipids, APOE, and several other possible stimuli at the plasma membrane by acting as a crucial component of the microglia sensitizer (12, 13). Tyro protein kinase binding protein (TYROBP/DAP12), a junctional protein that activates downstream signaling cascades, including SYK, ERK, PLCG2, and NFAT, is necessary for TREM2 to transduce the intracellular response. While TREM2 mutations causing total loss of function are closely linked to frontotemporal lobe dementia and Nasu-Hakola disease, AD-associated TREM2 mutations (such as R47H and R62H) are believed to cause partial loss of operation because they occur within the ligand-binding domain (14). Although this link is unclear, some investigations have shown that two loss-of-function TREM2 mutations, Q33X and W191X, may similarly alter AD risk (15). Nonetheless, research on TREM2 deletions has significantly improved our knowledge of TREM2 activity and uncovered significant distinctions between human and mouse cells at the late and early stages of illness (16, 17). It is critical to look into TREM2’s role in human microglia to comprehend better these characteristics and how they affect human illnesses (18). Although scRNA-seq technology was used in this study to understand AD heterogeneity and key cell populations in microglia-specific AD risk loci, it is now necessary to combine scRNA-seq with new computational frameworks or particular technologies to better understand its pathophysiology and molecular regulatory mechanisms, which may help to develop better AD treatments.

Therefore, we investigated the expression characteristics and Possible regulatory mechanisms of immune-related genes in AD by combining single-cell scRNA-seq data with bioinformatics analysis of RNA-seq data. In addition, we predicted promising therapeutic Chinese medicines through the database. We constructed a “Chinese medicine-ingredient-target” network to screen potential therapeutic targets, laying the foundation for developing RNA-based therapeutic strategies for AD.

## Materials and methods

### Downloading and processing of data

The single-cell transcriptome dataset GSE158234 was downloaded from the GEO database.^1^ The database contains 4 samples of Single-cell comparison of WT and KO TREM2 microglia isolated from 6 months-old WT and 5X-MITRG mice (18). It was also downloaded the 18 SORL1-WT samples and the 18 SORL1-KO samples RNA-seq dataset expression profiles GSE238013^2^ from the GEO database. We first use the R package “Seurat” (19) to download the original data for quality control and filtering. For the expression spectrum data set, we obtain the observation information of the probe, map the investigation to the gene, remove multiple matches, and when various examinations match a gene, use the mean value as the gene expression, and finally get the gene expression spectrum. Additionally, AD-related genes were obtained from the DisGeNET database,^3^ the Comparative Toxicogenomics Database,^4^ the Genecards database^5^ and the OMIM database.^6^ We selected a group of genes shared by four databases as highly reliable AD-related genes.

### Dimensionality reduction analysis of single-cell data and identification of cell types

To obtain a reliable cell subgroup, we use the R package “Seurat” (19) to filter single-cell data for data processing, setting each gene to be expressed in at least 3 cells, each cell expressing at least 250 genes. By using the PercentFeatureSet function, we calculate the percentage of molecules and rRNA and make sure that every cell expresses >500 genes and <4,000 genes, with a molecule content of <30%, with at least 100 unique molecular identifiers (UMI) in each cell. Then, the data is naturalized, and highly variable genes are found using the FindVariableFeatures function. Finally, the cells are clustered (resolution = 0.1, dim = 30) using the FindNeighbors and FindClusters functions and visualized by the t-distribution random neighbor embedding (t-SNE). The marker genes for each cell are identified by the FindAllMarms function (logfc = 0.5, Minpct = 0.35) to determine the cell type of each cell.

### Critical gene identification and protein–protein interaction network analysis

The numerous ways that intersecting genes affect AD at the systemic level were discovered using the R package “WebGestalt” (20) to evaluate potential important gene sets for GO biological process (BP), cellular component (CC), molecular function (MF), and KEGG pathway enrichment. With the significance level set to *p* < 0.05, we displayed the data using the “ggplot2” package.

Using Cytoscape, we performed a network analysis based on modules (21). To get network modules, we used the MCODE plugin (22), which detects closely linked protein clusters in the target network, with “degree cutoff = 2, maximum. Depth = 100, k-core = 2, and a cutoff value of node score = 0.2.” We obtained information on gene interactions from the String database,^7^ and we then used the plugin to identify protein clusters with a high degree of connectivity in the target network.

### Mapping of cell subgroups

Reassess RNA-seq expression spectral data sets using the CIBERSORT algorithm (23), which uses the expression of labeled genes in each cellular subpopulation as a background for figuring out the ingredients of specific cellular subpopulations in various samples of the expression profile. The R package “Hmisc” was used to calculate further Pearson correlation coefficients and the importance of gene expression in connection to immune cells, and the R package “ComplexHeatmap” was used to show the results (24). Pearson correlation analysis was used to assess the differences between the WT and KO groups, and the significantly expressed genes in the KO group were ultimately chosen as hub genes. Pearson correlation analysis determined the importance of AD-related genes with immune cells. Examining the degree of correlation between two or more variables is one of the most widely used statistical procedures (25). The product-moment correlation coefficient, also known as the Pearson correlation coefficient (*r*), is one of the most commonly used statistical data (26). Correlation methods have been used in various situations in medical research. First, to determine whether a statistically significant positive or negative correlation exists between two or more variables. Second, the degree of statistical significance of the correlation is measured. Third, to determine the proportion of variability in the dependent variable (*X*) that can be explained or “accounted for” by the independent variable (*Y*), and fourth, to test the goodness-of-fit of linear regression (25). The correlation method is used in a variety of situations.

### Hub genes’ GSEA enrichment analysis and ROC curve construction

The GeneMANIA database^8^ is used to build networks of protein–protein interactions (PPIs) (27). The database generates gene function hypotheses through functional analysis, rates gene lists, and ranks genes in order of importance. By identifying functionally linked genes and classifying them according to expected values based on genome-wide and proteomic data, core gene networks for mechanistic study may be created.

The structure of the nomogram is effective for identifying clinical AD. The R package “rms” was used to determine the prediction efficacy based on candidate genes (28). Overall, we obtained the expression profiles of each hub gene in RNA-seq profiling dataset GSE238013,The PROC package was used to evaluate the diagnostic predictive value of pivotal genes. Receiver operating characteristic (ROC) area under curve (AUC) and 95% confidence intervals (CI) were calculated to illustrate the diagnostic usefulness of AD testing and assessment of candidate genes. AUC >0.7 was regarded as the most straightforward diagnostic cost (29).

### Traditional Chinese medicine prediction of probable therapeutic benefits

The traditional Chinese medicine (TCM) with potential interventional effects were identified using the Coremine Medical database,^9^ Standard screening with *p* < 0.05, and further screening based on the theoretical knowledge of TCM and the principle of everyday clinical use if the quantity of the drug is excessive (30). The active ingredients of the anticipated TCM were screened from the TCMSP database^10^ based on bioavailability (OB) ≥30% and drug-like properties (DL) ≥0.18, and the corresponding compounds’ targets of action were obtained. The UniProt database^11^ was used to de-weight and normalize the valid targets, and the de-weighted and normalized data were then imported into Cytoscape to create a network diagram of “Traditional Chinese Medicine-Ingredient-Target.” The top 20 targets were then screened and visualized using the degree algorithm in the CytoHubba plugin (31).

### Random Mendelianization

Mendel’s randomization uses only data that is available in an open database. We define SNP as IV and apply double-sample MR to investigate the causal link between the hub gene and AD risk. Hub gene information from a public GWAS data source. As a representative gene for Alzheimer’s disease, we chose the most important gene, IL1B, and conducted a Mendel randomized study. Data on IL1B can be obtained at https://gwas.mrcieu.ac.uk/datasets/?trait__icontains=interleukin-1%20beta, and data on AD can be accessed at http://gwat.mrceu. Ac. Ak/datesets/?Trait__ICONTAINS=Alzheimer's%27s%20disease. Reverse differential weighting (IVW) was used to assess the connection between hub gene levels and AD risk in the MR analysis based on the R package “TwoSampleMR.” MR-Egger was used in further sensitivity studies (32).

## Results

### Clustering and dimensionality reduction analysis

**Figure 1:**
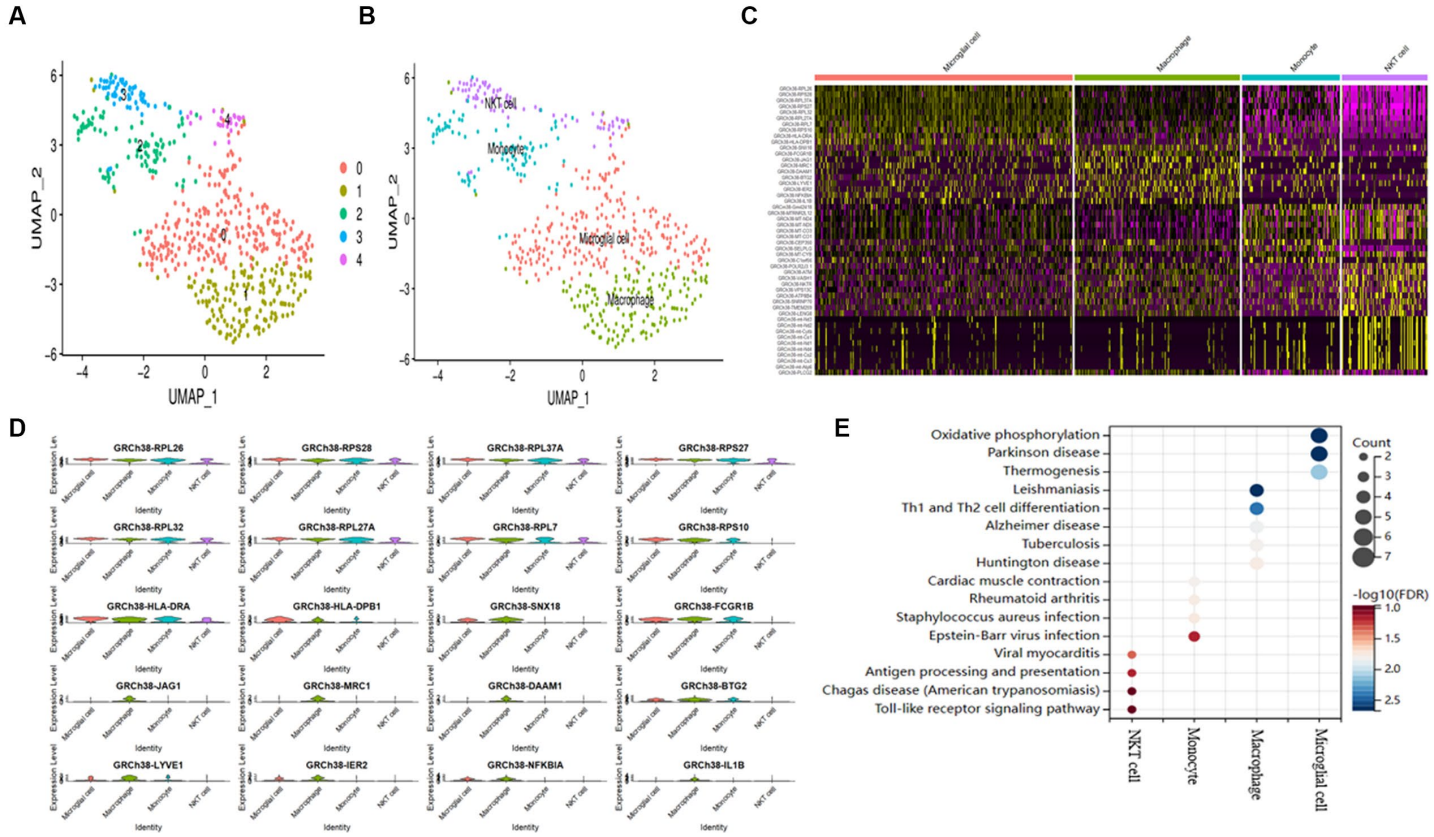
AD samples of single-cell sequencing. **(A)** T-SNE plots of different subpopulations of 5 cell clusters. **(B)** The maker genes identify four cell types. **(C)** Heatmap showing the top 10 genes in 4 expressed cell types. **(D)** Expression of the top gene in different cell types. **(E)** Examining the KEGG enrichment of the four cell types.

Supplementary Figure S1 describes the process diagram of the bioinformatics analysis of the study. Single-cell filtering and percentage-collection functions generate 1,210 genes. In Supplementary Figure S2A, the amounts of UMI and mRNA are significantly correlated. After quality control, the sample’s mRNS/UMI/polymeric /rRNA content is evenly distributed (Supplementary Figure S2B). We operate the data in a de-weight operation to ensure the effectiveness and feasibility of follow-up analysis. First, identify the high-variable genes in cells (which are interfering significantly in each cell), follow up only the highly modified genes that contribute to the dimension of the cell cluster, and only retain the contributing PCs for the identification of cell subgroups (Supplementary Figure S2C). Use the “ScaleData” function to scale all the genes taken from the scRNA-seq dataset GSE158234, then use PCA degradation to find the anchor point (Supplementary Figure S2D). Using the “FindNeighbors” and “FindClusters” algorithms, a cluster analysis of 1,210 genes revealed five clusters (Figure 1A). The maker gene divided These five clusters into four cell types (Figure 1B). The expression of the marker genes in the four cell types was discovered after screening the marker genes of the four cell types using the “FindAllMarkers” program (Figures 1C,D). KEGG enrichment showed that the four cell subtypes discovered using scRNA-seq study had significant heterogeneity (Figure 1E).

### Screening of cell subpopulations

**Figure 2:**
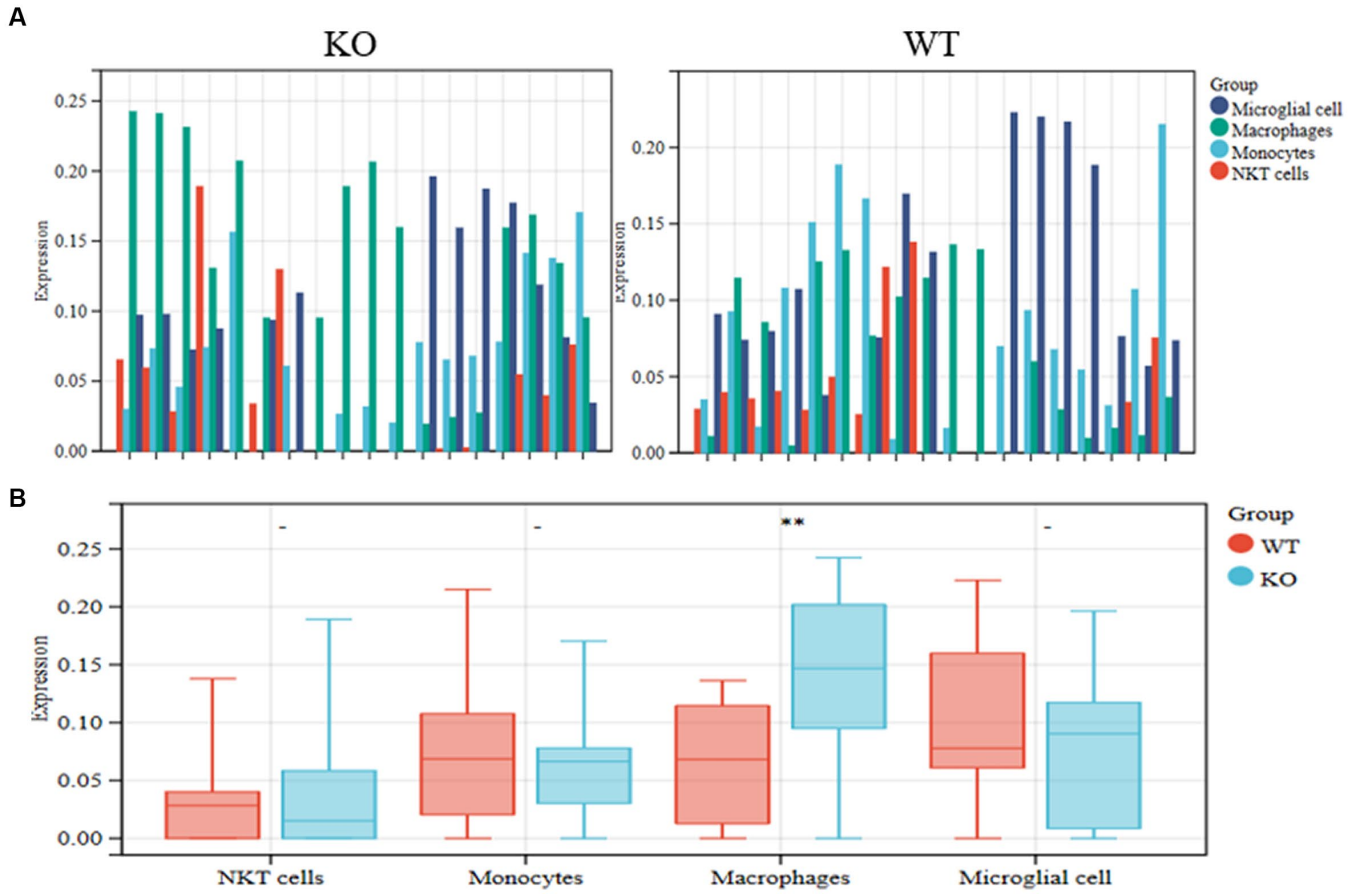
Identification of differential cell subpopulations in RNA-seq datasets. **(A)** The KO and WT groups’ relative abundance of the identified cell types was calculated using the CIBERSORT method on the GSE238013 dataset. **(B)** Statistical diagrams of the four discovered cell types. ^**^p < 0.01.

To search for subgroups of AD patient differences further, we assessed the number of identified cell types in the KO and WT groups from the GSE238013 dataset using the CIBERSORT approach (Figure 2A). The results showed that the frequency of macrophages in the KO group was more significant than that of the WT group (Figure 2B).

### The discovery of genes associated with AD

**Figure 3:**
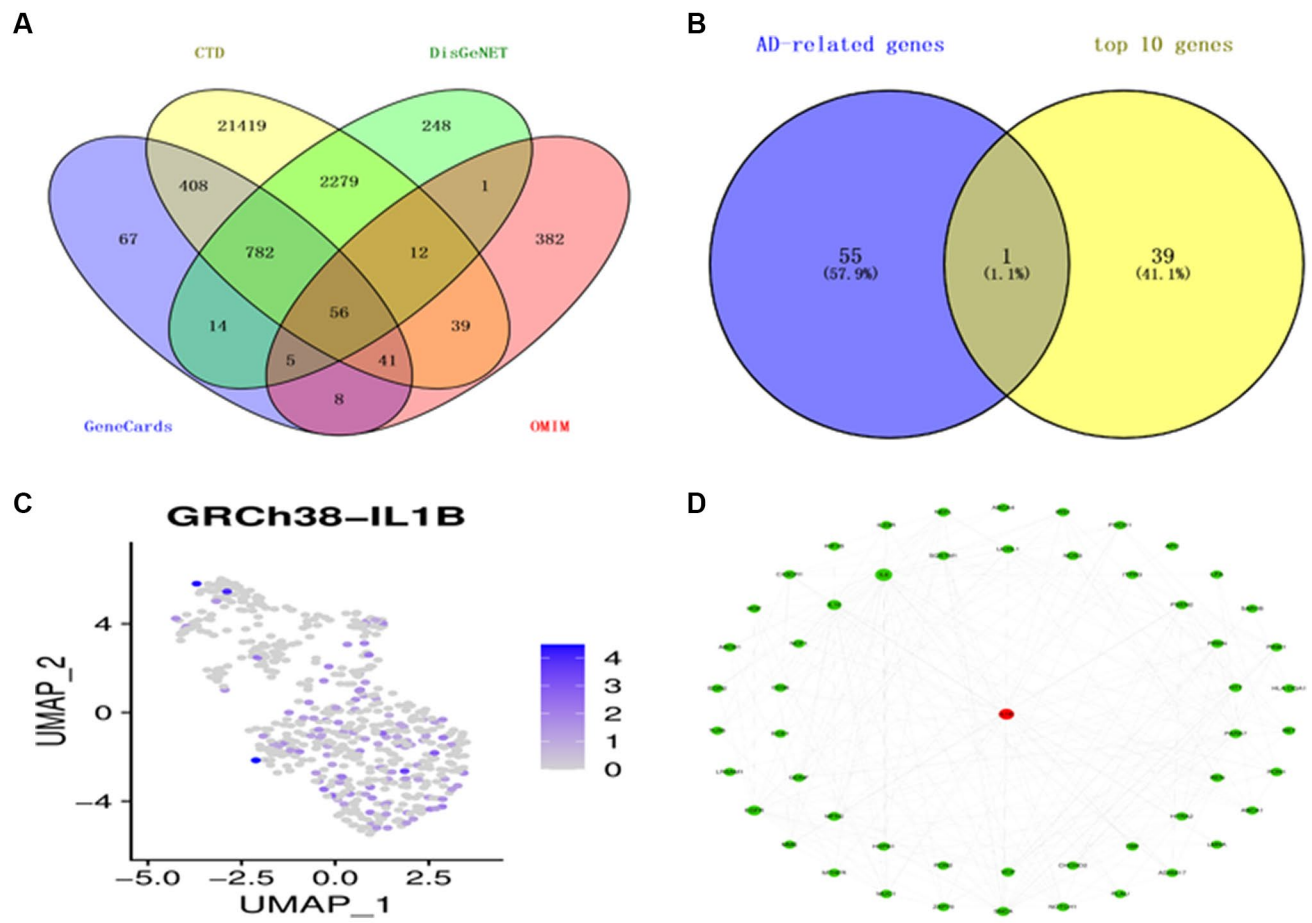
Identification of AD-related genes. **(A)** A Venn diagram illustrates four datasets’ gene intersections related to AD. **(B)** The top 10 genes from each cell type are shown in a Venn diagram as genes related to AD. **(C)** T-distribution-based random embedding for IL1B. **(D)** The protein–protein interaction (PPI) network for 54 genes linked to AD.

Utilizing overlap analysis to look for genes linked to AD in the DisGeNET, CTD, Genecards, and OMIM databases, we found 56 overlapping genes (Figure 3A). The top 10 genes of 4 distinct cell types and the shared genes for AD were intersected to provide a significant gene, IL1B, classified as a macrophage (Figure 3B). The expression level of the gene IL1B in a single cell is shown in Figure 3C. According to the PPI analysis utilizing the String database, there were 54 common genes for the subsequent investigation (Figure 3D).

### Functional study of genes associated with AD

**Figure 4:**
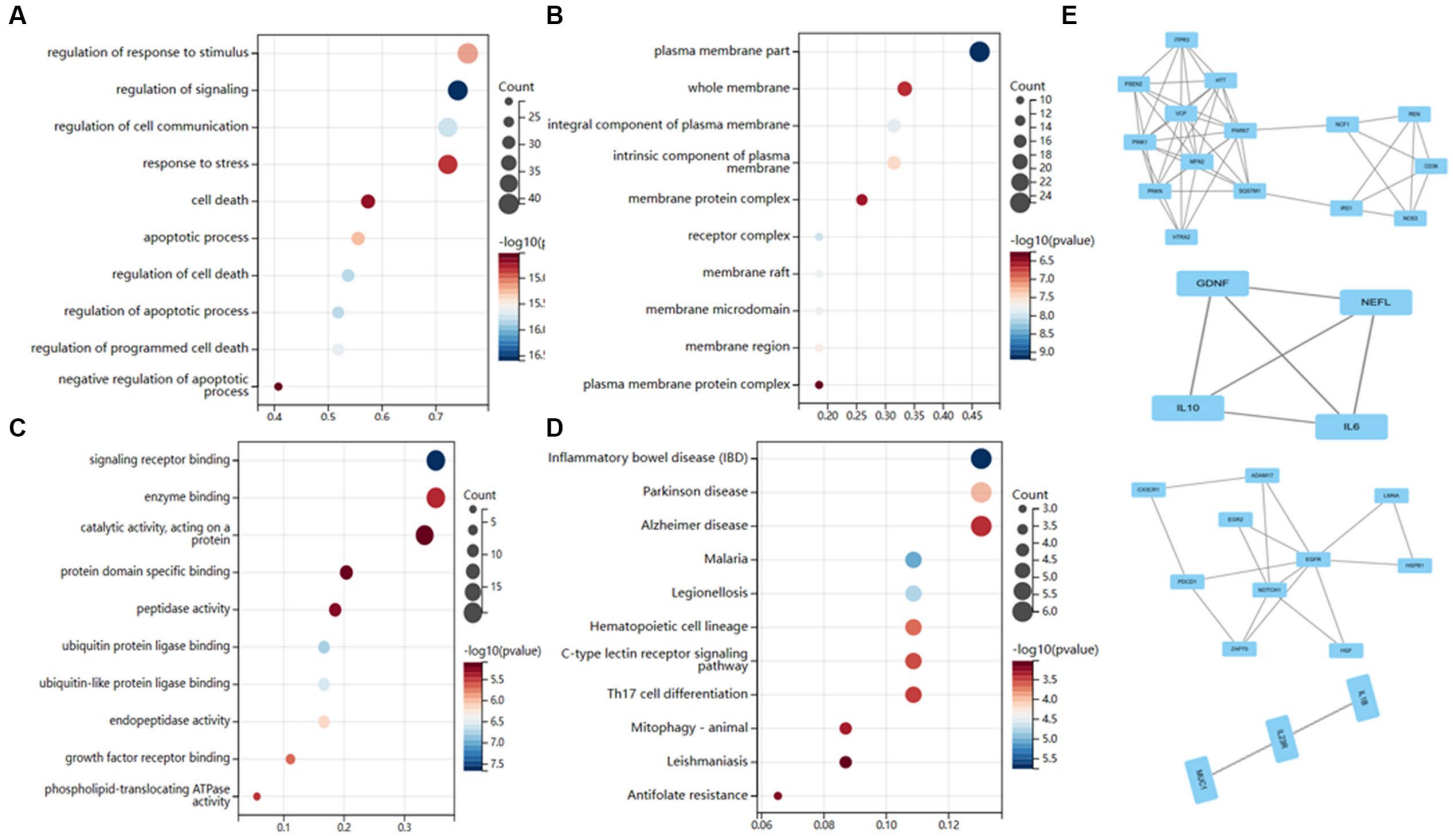
Functional enrichment analysis of AD-related genes. **(A)** Biological processes enrichment. **(B)** Cellular component enrichment. **(C)** Molecular function enrichment. **(D)** KEGG pathway enrichment. **(E)** There were a total of 4 crucial clusters in the MCODE-based network.

We conducted KEGG and GO enrichment analyses to explore these genes’ functional notes. BP enriches 1,000 terms (*p* < 0.05) for the GO functional notes of genes, with the result of the top 10 notes as shown in Figure 4A.CC is rich in 356 terms, with the results of the top 10 notes as shown in Figure 4B. Three hundred eighty-nine terms are abundant in MF, with the top 10 notes, as shown in Figure 4C. The analysis of the KEGG path enrichment shows that 181 paths have been enriched, with the top 10 notes as shown in Figure 4D.

Cytoscape also examined module-based networks and used mature MCODE algorithms to locate module-based network protein clusters in the target network. The results showed that the MCODE algorithm obtained 4 clusters (Figure 4E).

### Using a web-based computational framework for pharmacology, identify the hub genes

**Figure 5:**
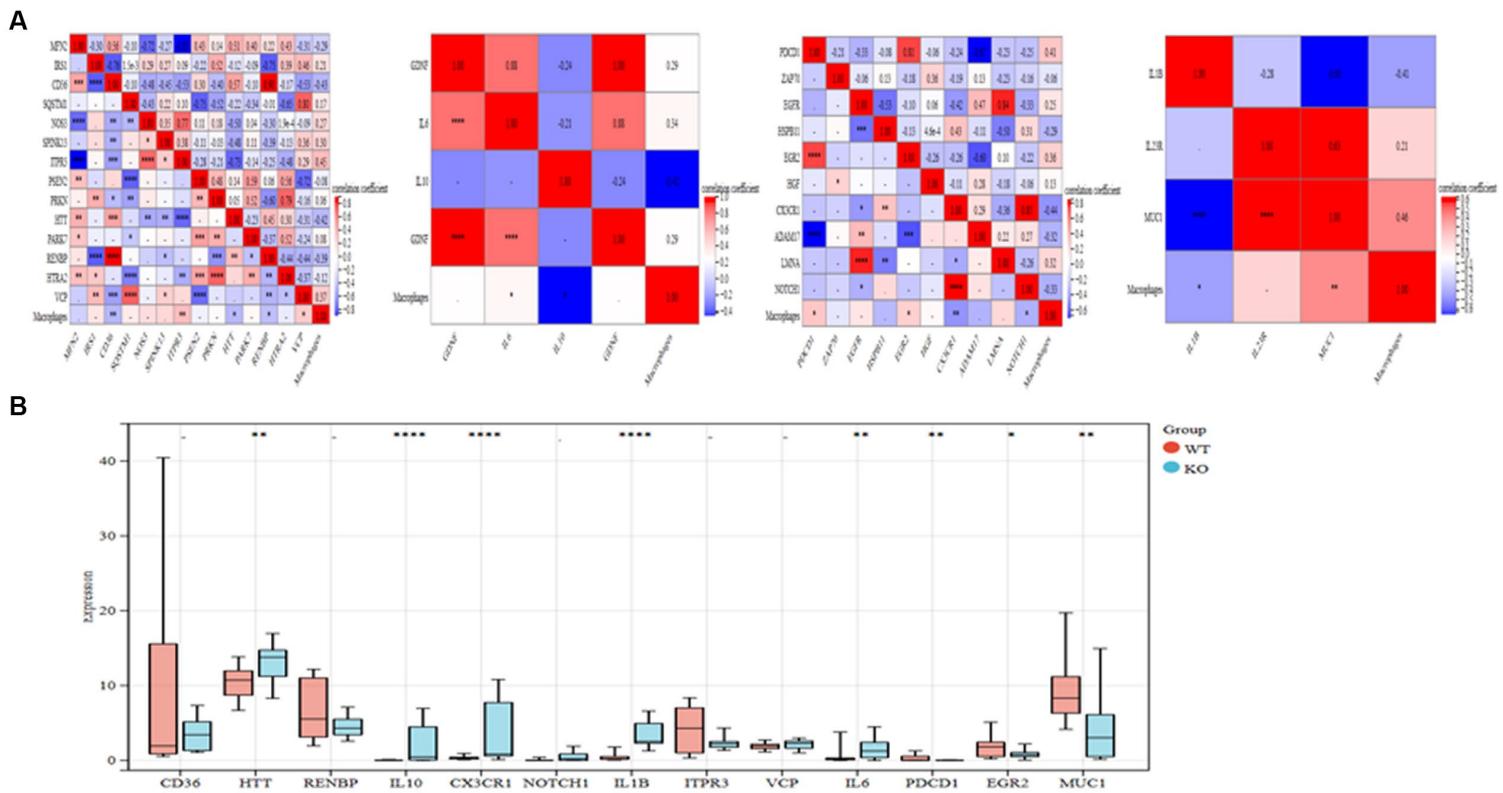
Important genes in the core cluster are identified. **(A)** Study of four gene clusters and macrophages in correlation. **(B)** The bar graph displays 13 significantly linked genes’ differential expressions in the WT and KO groups. ^*^p < 0.05, ^**^p < 0.01, and ^****^p < 0.001.

Based on the MCODE algorithm, four clusters were obtained using the R package “Hmisc” to analyze the correlation of the genes with the macrophages in the four clusters. Six genes in the four clusters (ITPR3, VCP, IL6, PDCD1, EGR2, MUC1) are significantly positive correlation to macrophages. In comparison, 7 genes (CD36, HTT, RENBP, IL10, CX3CR1, NOTCH1, IL1B) are significantly negative correlation to macrophages (Figure 5A). Five highly expressed genes (HTT, IL10, CX3CR1, IL1B, and IL6) from the KO group are ultimately selected for further study after bar diagrams are used to demonstrate the differential expression of these 13 highly associated genes in the WT and KO groups (Figure 5B). The linkages between the five interact genes were discovered using the String database. As can be observed, four genes (IL10, CX3CR1, IL1B, and IL6) that are directly regulated by one another function as AD hub genes (Supplementary Figure S1).

### GeneMANIA, GSEA enrichment analysis, and diagnostic value assessment

**Figure 6:**
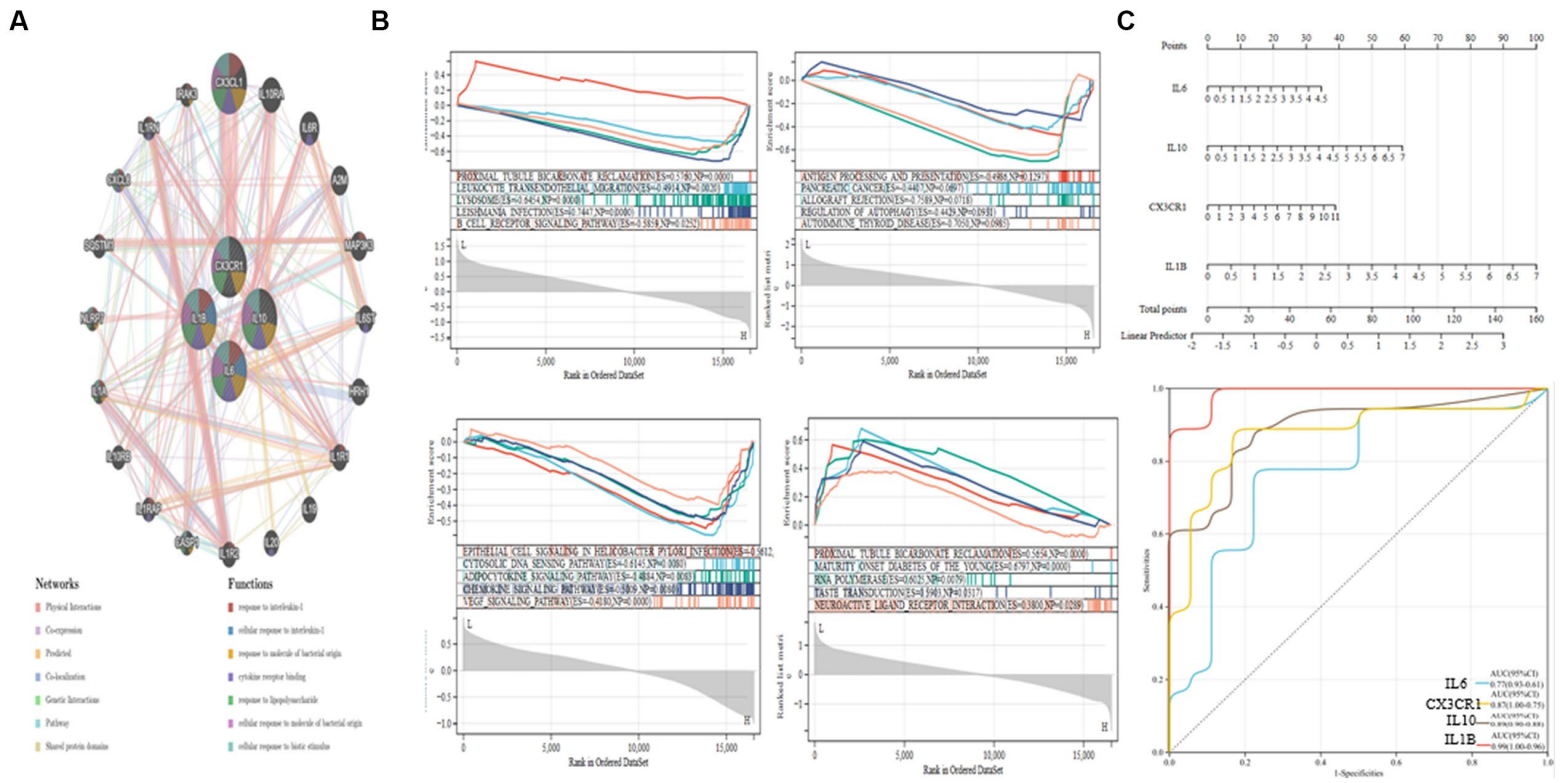
GSEA analysis and ROC curve of hub genes. **(A)** PPI network establishment. **(B)** IL10, CX3CR1, IL1B, and IL6 GSEA analysis. **(C)** Nomogram and ROC curves for every hub gene.

The PPI network and the connections of IL10, CX3CR1, IL1B, and IL6 are shown in Figure 6A. According to the GeneMANIA study, these pathways are closely associated with the immunological inflammatory response. The enrichment scores of each gene in several KEGG pathways were also examined using ssGSEA, and the findings indicate that most of them have direct regulatory linkages (Figure 6B).

Next, we built a Nomogram to support four possible center genes (Figure 6C). We created a ROC curve to assess the specificity and sensitivity of each gene used to diagnose the disease. It is followed by IL1B (AUC 0.99, CI 0.96–1.00), IL10 (AUC 0.89, CI 0.88–0.90), CX3CR1 (AUC 0.87, CI 0.75–1.00) and IL6 (AUC 0.77, CI 0.61–0.93). The results showed that all hub genes had potential diagnostic value for AD (Figure 6C).

### TCM prediction of probable therapeutic benefits

**Figure 7:**
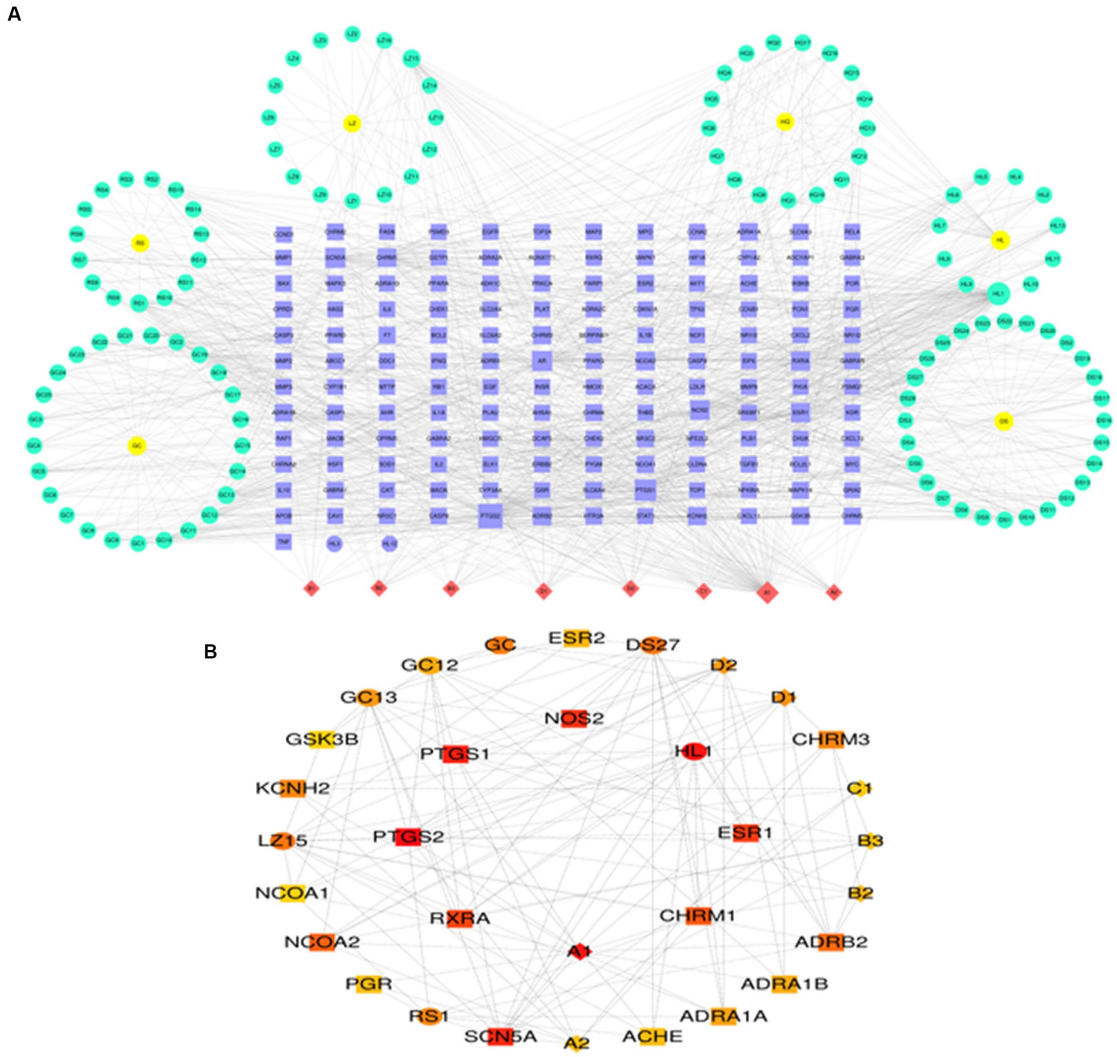
Prediction of potential therapeutic TCM related to AD. **(A)** TCM-ingredient-target network diagram. **(B)** Predicting key targets for TCM intervention in AD progression.

Supplementary Table S1 demonstrates that to find prospective therapeutic TCM, four genes were connected to the Coremine Medical database (see footnote 9). According to a study of Chinese medical theory, *Salvia miltiorrhiza*, *Ganoderma lucidum*, and ginseng have several effects, including enhancing blood circulation, reducing blood lipids and antioxidants, accelerating blood circulation, and removing blood stasis. *Glycyrrhiza glabra* can strengthen the spleen, enhance qi, and efficiently expel heat and remove poisons. *Scutellaria baicalensis* and Rhizoma coptidis help dry moisture and diarrhea and detoxify and eliminate heat. The above TCMs are often used in the Chinese medicine clinic to treat AD for subsequent analysis (33–38). The active ingredients and targets for the six traditional Chinese medications indicated above were discovered using the TCMSP database. Altogether, 3,259 targets and 328 active compounds were found. In the Uniprot database, the targets of the components mentioned above were genetically de-emphasized and normalized, and a network diagram of “Chinese medicine-component-target” was created (Figure 7A). Using the CytoHubba Plugin “Degree” algorithm, marks of the first 30 are screened and presented in different color stratifications. The active ingredients of the above 6 TCMs are mainly involved in the regulation of prostaglandin-endoperoxide synthase 1 (PTGS1), prostaglandin-endoperoxide Synthase 2 (PT GS2), retinoid X receptor, alpha (RXRA), cholinergic receptor and other genes such as muscarinic 1 (CHRM1), nitric oxide synthases 2 (NOS2), estrogen receptor 1 (ESR1), which may be a potential drug to interfere with the pathological development of AD (Figure 7B).

### The risk of AD is causally related to IL1B

**Figure 8:**
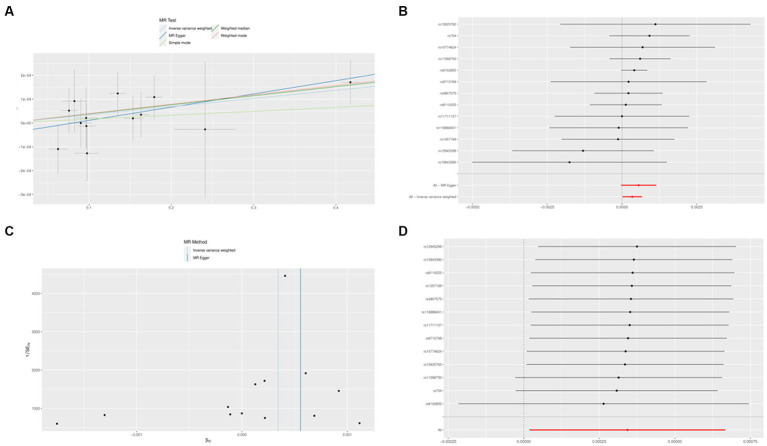
The Mendelian randomization experiment results. **(A)** A scatterplot demonstrating how IL1B raises the likelihood of AD. **(B)** A forest plot showing the causal relationship between each SNP and the risk of AD. **(C)** A funnel plot shows the overall variability of the MR assessments of the effect of IL1B on AD. **(D)** A leave-one-out figure showing how IL1B and the risk of AD are related causally.

No SNP was a weak instrumental variable in the studies taken into inclusion. The causal effect of each SNP variation on AD is shown in Figures 8A,B. We investigated the relationship between IL1B levels and AD. Using the IVW method, we found that IL1B was associated with a higher risk of AD, with an OR of 1.003 (95% CI = 1.001–1.006, *p* = 0.038). The MR-Egger method could not show significant statistical significance (OR = 1.005, 95% CI = 0.999–1.011, *p* = 0.091). With the margin of MR Egger’s regression not detecting horizontal pluralism (*p* = 0.942) and the Funnel diagram’s causal impact being broadly symmetrical, further indicating that pluralism does not have biased causal effects (Figure 8C). Following the systematic removal of each SNP, as shown in Figure 8D, we once again conducted MR analysis on the remaining SNPs. The findings were consistent, indicating a robust causal connection between the outcomes of all SNP computations. Additionally, this suggests that the IL1B level and AD do not include any apparent SNPs and that the validity of earlier MR findings is maintained.

## Discussion

Traditional molecular biology investigations have partly uncovered the pathogenic underpinnings of AD in earlier studies, but further research is required. The development of scRNA-seq technology can provide new insights into the pathological development process of AD. This study found four major cell types in the AD model induced by TREM2-KO (CRISPR-modified TREM2) induction: mononuclear cells, macrophages, microglia cells, and NKT cells. Notably, in the expression of the AD model induced by CRISPR modification to eliminate the SORL1 gene (SORL1-KO), the proportion of macrophages in the KO sample is significantly higher than in the WT sample. One study (39) showed that the absence of SORL1, as well as mutations of the AD gene of joint chromosome appearance, APP, in the amygdala protein precursor protein and PSEN1/2, caused the disease to converge through early endocrine expansion, which is a marker of the cellular pathology of AD. SORL1 acts in conjunction with the reverse recording transport complex, which can regulate the recovery of APP from the endosome, thereby causing endosome swelling and APP error processing, suggesting that one of the roles of SARL1 is to promote the endorphins degradation and clearance pathway of neurons. As highly phagocytic cells, Peripheral macrophages can also migrate to the brain in cultivation cells and animal models and have a higher ability to remove the Aβ fibril (40, 41). Simard created a chimeric mouse model by irradiate-treating APP/PS1 mice and injecting bone marrow cells into their blood. They then utilized this model to demonstrate that peripheral macrophages, as opposed to microglia, were more effective in removing Aβ fibril phagocytosis (42). Therefore, increasing macrophage cells is essential to eliminating AB fiber in AD. We investigated AD-related gene targets. We discovered that IL1B was highly expressed in macrophages and had significant interactions with several genes relevant to AD, suggesting that IL1B could be the crucial gene. We developed an IL1B-based disease-specific regulatory network for a set of AD-related genes. GO functional enrichment study showed that these genes influence signaling receptor binding, enzyme binding catalysis, response to stimuli, plasma membrane component, and other GO enrichment pathways. We discovered substantial gene–gene interaction. These genes are also prevalent in many signaling pathways, including those connected to inflammatory bowel disease (IBD), Parkinson’s disease, and Alzheimer’s disease. These results suggest that these genes regulate several essential and complex disease processes.

Four modules were created from the genes using network module analysis. The four modules were investigated for connections to macrophages. Seven genes (CD36, HTT, RENBP, IL10, CX3CR1, NOTCH1, IL1B) were significant negative correlation associated with macrophages, while six of them (ITPR3, VCP, IL6, PDCD1, EGR2, MUC1) were substantial positive correlation connected with macrophages. Five genes (HTT, IL10, CX3CR1, IL1B, and IL6) differently expressed in the KO group were discovered after the significant association genes were further differentially expressed in the WT and KO groups. Four of these genes (IL10, CX3CR1, IL1B, and IL6) were chosen as the AD hub genes since it was found via the string database that they had a direct regulatory link. Interleukin (IL)-10 is a crucial immunomodulatory cytokine with pleiotropic immunosuppressive properties that can be produced by a variety of leukocyte subpopulations and is influenced by these subpopulations’ effects on signaling pathways and transcriptional networks (43). Depending on the expression of its receptor, IL-10 has anti-inflammatory solid effects on different immune system cells (44). The generation of antibodies by B cells has been found to increase in response to interleukin-6 (IL-6), a 26-kD secretory protein. Later, it was shown that IL-6 collaborates on several tasks with other cytokines in the IL-6 family via the same IL-6 signaling transducer, gp130. Cells are activated in various ways by IL-6 receptors on membranes and in soluble form (45). Interleukins are released by leukocytes and activated microglia. Macrophage and neutrophil activity are stimulated by interleukin, which also promotes T-lymphocyte-mediated toxicity. It has several receptors, including the IL-6 receptor, which promotes neuronal cell survival at ordinary concentrations but may cause neurodegeneration at high levels. Mutations in IL-6 may raise the risk of AD. Additional receptors include IL1B, IL-9, IL-17, IL-15, and IL-10. By inhibiting the activity of several proinflammatory mediators, IL-10 acts as an anti-inflammatory molecule. IL-18 sparks interferon-gamma synthesis. Each of them contributes in some way to the neuroinflammation that leads to AD, either directly by producing neurotoxicity or indirectly by activating inflammatory mediators (46). Chemokines are chemotactic cytokines that mediate AD and encourage chemotaxis in the brain. Chemokines are classified into four families: CXC, CC, CX3C, and C. One of the many crucial glial cell communication axes that maintain microglia homeostasis is CX3CR1 and its neuronal ligand CX3CL1 signaling, and loss-of-function variants of CX3CR1 have been associated with worsening Braak staging, neurodegeneration, as well as decreased survival in patients with AD and ALS (47). Microglia and macrophages gather in the vicinity of amyloid plaques. These activated cells release cytokines, including interleukin, which have proinflammatory properties and hasten the neuroinflammatory processes in the brain that lead to AD (48). Next, we built a Nomogram to support four possible center genes and created an ROC curve to assess the specificity and sensitivity of each gene used to diagnose the disease. The results showed that all hub genes have potential diagnostic value for AD.

Since pro-inflammatory cytokines and their receptors are the leading cause of neuroinflammation in AD, inhibiting cytokine gene expression and blocking or binding to their receptors could be a better therapeutic option for treating AD. TCM has various active components that may act on multiple targets simultaneously and provide synergistic therapy in AD patients, considering the complicated and multifactorial pathophysiology of the illness, making it safer than synthetic drugs with single-target action (49). In light of this, six Chinese medicines, Including Radix Salviae, ginseng, Ganoderma, licorice, Coptidis Rhizoma, and Scutellariae Radix, were included in the Coremine Medical database. A network pharmacological study was carried out to investigate further the mechanism of action of these six herbs in treating AD. It was shown that these herbs functioned mainly by modulating the genes of PTGS1, PTGS2, RXRA, CHRM1, NOS2, and ESR1. In a study by Chong et al. (45), six Radix Salviae components were examined to see how they affected these critical aspects of AD. The results showed that each ingredient might lessen A’s toxicity. In addition to reducing A aggregation, tanshinones I, IA, IIA, and crypto tanshinone all exhibit protective effectiveness against A-induced cellular damage. When comparing resistance to tanshinic acid A-induced cytotoxicity and resistance to A-aggregation, crypto tanshinone and tanshinic acid A showed comparable working concentrations. This observation implies that the capacity of tannic acid A and cryptotanshinone to inhibit A-aggregation is the primary cause of their protective effects against A-induced cytotoxicity. The main cytokine produced by Th17 cells, IL-17, attaches to its receptor, activates signaling pathways inside cells, and causes the release of many proinflammatory molecules, including IL-6, which exacerbates neuroinflammation (50). The JAK/STAT system contributes to neuroinflammation in AD through excessive activation. When IL-6 activates JAK2 and JAK3, STAT is phosphorylated by JAK3, and apoptosis in the mitochondrial pathway is initiated, leading to mitochondrial dysfunction in AD (51). Ganoderic acid A may minimize the imbalance of the Th17 / Tregs axis and the neuroinflammatory effects in AD mice, according to Zhang’s et al. research (37). The fundamental mechanism involves the JAK/STAT signaling pathway, which Th17 cells suppress. Many of the active components found in ginseng, including ginsenosides, polysaccharides, amino acids, and polyacetylene, have therapeutic benefits in treating AD, according to contemporary medical studies. In China, ginseng has been used to treat dementia for thousands of years. Ginsenosides have been shown to alter synaptic plasticity and the cholinergic system, reduce A exacerbation and tau hyperphosphorylation, and have anti-neuroinflammatory, antioxidant, and anti-apoptotic properties in the treatment of dementia, among other things, by Wang et al. (36). Additionally valuable for treating AD are ginsenosides, oligosaccharides, polysaccharides, and ginseng proteins found in ginseng. Licorice reduces microglia activation and inflammation in lipopolysaccharide (LPS)-induced neurotoxicity via inhibiting the AP1 and NF-kB pathways. When neurological diseases like Alzheimer’s are accompanied by inflammation, this inhibition halts the neurotoxic process (52). Additionally, it has been shown that licorice improves *in vivo* cognitive signs of Alzheimer’s disease. Licorice has been shown to have anticholinesterase properties that may counteract the amnesic effects of scopolamine and diazepam. Anticholinesterase medicines often treat Alzheimer’s disease (53). According to Jin’s et al. research (34), baicalin reportedly prevented microglia from activating, decreased neuroinflammation, and postponed neuronal loss; inhibition of NLRP3 inflammatory vesicles and blocking of the TLR4/NF-B signaling pathway may also play a role in baicalin’s therapeutic action on AD. To investigate changes in sphingolipid metabolism in the brains of APP/PS1 mice following Huanglian changes in APP/PS1 rats’ cerebrospinal fluid after HLJDD treatment and in BV2 microglia triggered by A25-35. The findings showed that HLJDD significantly improved abnormal sphingolipid metabolism *in vivo* and *in vitro*. As indicated, the results show that the study’s conclusions on drug prediction are reliable, compatible with actual clinical situations, and consistent with TCM’s knowledge of AD. However, specific mechanisms remain unclear and need further research. The present study indicated that TCM would affect PTGS1, PTGS2, RXRA, CHRM1, and NOS2 targets via network pharmacology to prevent and regulate AD, which might provide a beneficial path for future investigations on TCM in AD. However, the results from bioinformatics analysis alone are far from satisfactory and must be confirmed by several studies.

Finally, using a two-sample MR analysis based on several GWAS data on IL1B (exposure) and AD (outcome), we looked at studies showing causal relationships between IL1B levels and AD risk. This MR study suggests that higher IL1B levels may be directly associated with an increased risk of AD. Although reverse causality and other systemic flaws that affect the results of traditional observational research are eliminated by MR, fundamentally, MR is similar to prospective randomized controlled trials (RCTs). The high precision of genotyping may successfully stop regression dilution caused by inaccurate testing. We only included patients from European populations to exclude potential confounding variables between IL1B and AD that the SNP could have brought about. To verify the stability of the results, we also performed MR-Egger regression tests, but we could not detect any evidence of directed-level pleiotropy.

## Conclusion

In summary, the present study describes the four cell types in AD through scRNA-seq analysis, integrates the systemic computational framework of network pharmacology and Single-cell sequencing, identifies the diagnosis genes of AD Interleukin and chemokines, and identifies potential TCMs and therapeutic targets that may interfere with the development of AD pathology, laying the basis for developing RNA-based AD therapy strategies.

## Data availability statement

The original contributions presented in the study are included in the article/Supplementary material, further inquiries can be directed to the corresponding authors.

## Author contributions

TW: Conceptualization, Data curation, Writing – original draft. XZ: Conceptualization, Data curation, Investigation, Writing – original draft, Methodology. WL: Formal analysis, Project administration, Writing – review & editing. FN: Methodology, Project administration, Writing – review & editing. XH: Data curation, Formal analysis, Writing – review & editing. LQ: Investigation, Methodology, Writing – review & editing. MC: Software, Supervision, Writing – review & editing. JY: Formal analysis, Investigation, Software, Writing – review & editing. SL: Validation, Visualization, Writing – review & editing. QW: Funding acquisition, Resources, Validation, Visualization, Writing – review & editing.

## Footnotes

^1^https://www.ncbi.nlm.nih.gov/geo/query/acc.cgi?acc=GSE158234

^2^https://www.ncbi.nlm.nih.gov/geo/query/acc.cgi?acc=GSE238013

^3^http://www.disgenet.org/

^4^http://ctdbase.org

^5^www.genecards.org

^6^https://www.omim.org

^7^https://string-db.org

^8^http://www.GeneMANIA.org

^9^https://www.coremine.com

^10^https://tcmsp-e.com/

^11^https://www.uniprot.org/
