# Supplementary material for: Identification of diagnostic molecules and potential traditional Chinese medicine components for Alzheimer’s disease by single cell RNA sequencing combined with a systematic framework for network pharmacology
Source: Front Med (Lausanne). 2024 Jan 5;10:1335512. doi: 10.3389/fmed.2023.1335512 (PMC10799563; doi:10.3389/fmed.2023.1335512)
Supplement: Supplementary file 1 [file Table_1.DOCX]

Supplementary Table 1 Prediction of TCM based on hub genes

| Hub genes | Predicting TCM |
| --- | --- |
| IL1B | Scutellariae Radix, Ginseng, Radix Salviae, Schisandrae Chinensis Fructus，Atractylodes Macrocephala Koidz. |
| IL6 | Ginseng,Scutellariae Radix，Radix Salviae, Forsythiae Fructus, Ginger peel |
| CX3CR1 | Uncariae Ramulus Cumuncis，Coptidis Rhizoma, Croci Stigma, Spirulina platensis |
| IL10 | Sonchus brachyotus, licorice, Ganoderma, Scutellariae Radix, Ginseng |
