# Supplementary figures and images for: Identification of diagnostic molecules and potential traditional Chinese medicine components for Alzheimer’s disease by single cell RNA sequencing combined with a systematic framework for network pharmacology
Source: Front Med (Lausanne). 2024 Jan 5;10:1335512. doi: 10.3389/fmed.2023.1335512 (PMC10799563; doi:10.3389/fmed.2023.1335512)

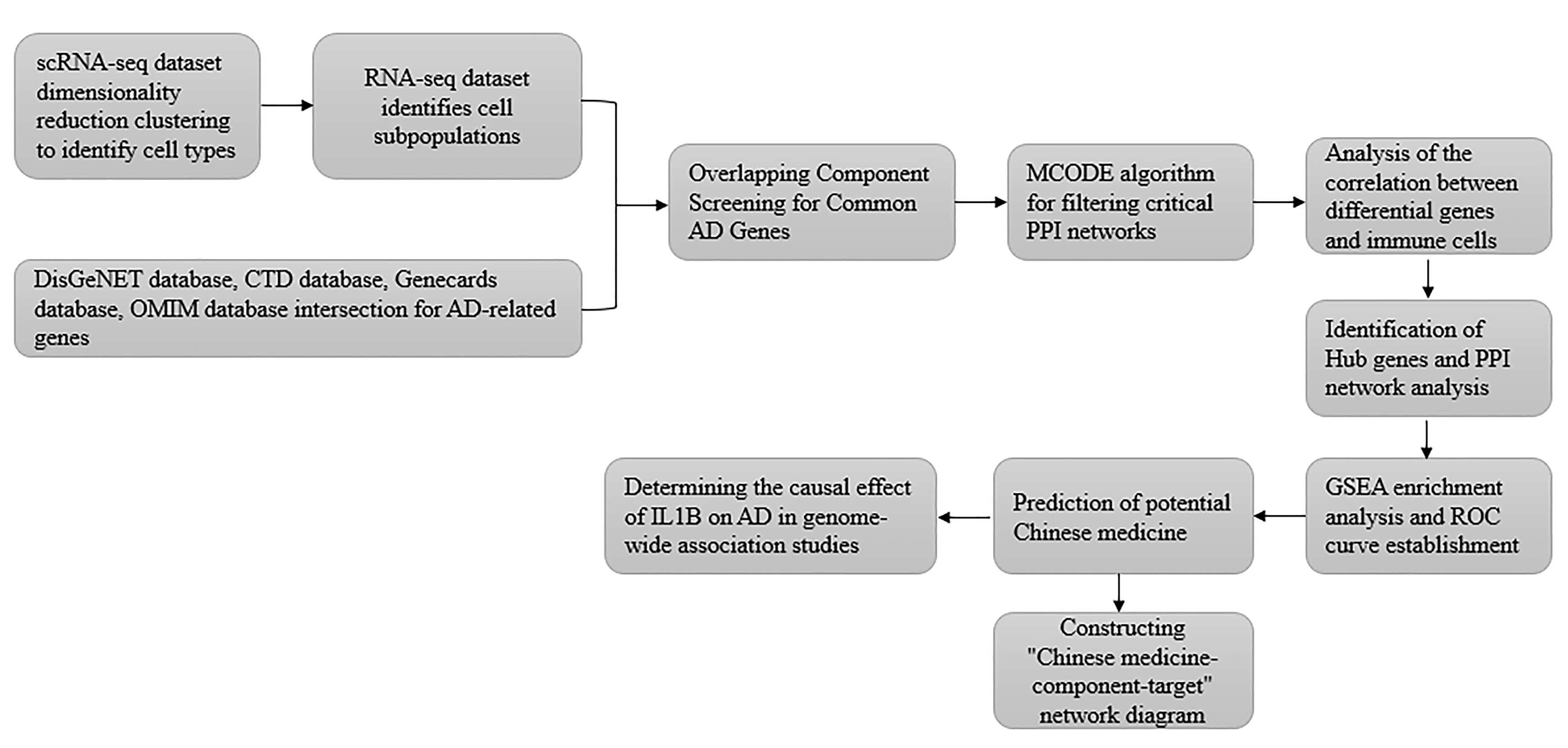

Supplement: Supplementary file 2 [file Image_1.TIF]

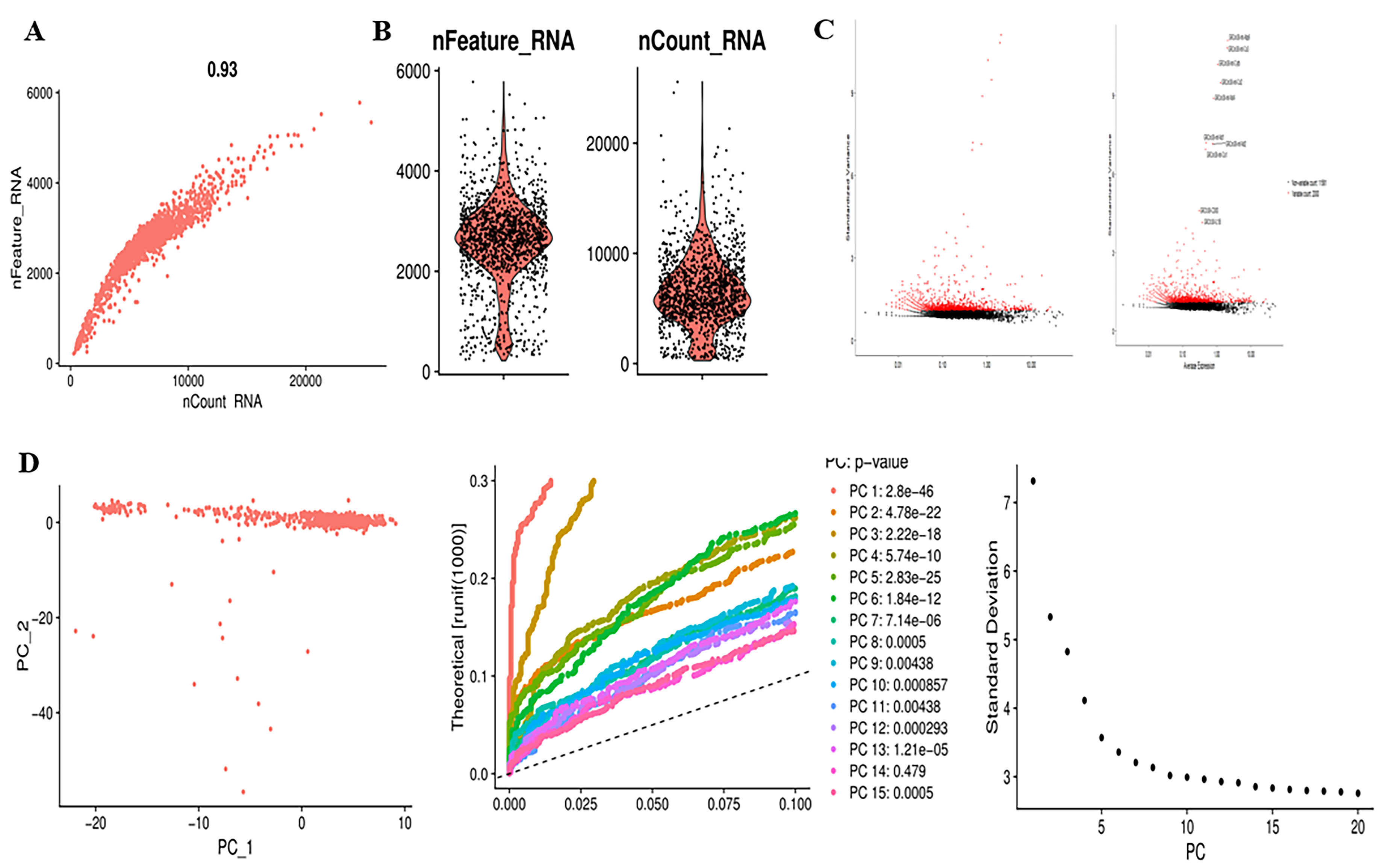

Supplement: Supplementary file 3 [file Image_2.TIF]
